# The Gilles De La Tourette Syndrome-Quality of Life Scale for Children and Adolescents (C&A-GTS-QOL): Development and Validation of the Italian Version

**DOI:** 10.3233/BEN-120274

**Published:** 2013

**Authors:** Andrea E. Cavanna, Chiara Luoni, Claudia Selvini, Rosanna Blangiardo, Clare M. Eddy, Paola R. Silvestri, Paola V. Calì, Stefano Seri, Umberto Balottin, Francesco Cardona, Renata Rizzo, Cristiano Termine

**Affiliations:** ^1^Michael Trimble Neuropsychiatry Research GroupBSMHFT and University of BirminghamBirminghamUK; ^2^Sobell Department of Motor Neuroscience and Movement DisordersInstitute of Neurology and University College LondonLondonUK; ^3^Child Neuropsychiatry UnitDepartment of Experimental MedicineUniversity of InsubriaVareseItaly; ^4^Department of Child Neurology and Psychiatry'La Sapienza' UniversityRomeItaly; ^5^Section of Child NeuropsychiatryDepartment of PediatricsUniversity of CataniaCataniaItaly; ^6^School of Life and Health SciencesAston Brain CentreAston UniversityBirminghamUK; ^7^Department of Child Neurology and PsychiatryIRCCS ’C. Mondino’ FoundationUniversity of PaviaPaviaItaly

**Keywords:** Gilles de la Tourette syndrome, tics, quality of life, wellbeing, behaviour

## Abstract

**Background:** Gilles de la Tourette syndrome (GTS) is a chronic childhood-onset neuropsychiatric disorder with a significant impact on patients’ health-related quality of life (HR-QOL). Cavanna et al. (Neurology 2008; 71: 1410–1416) developed and validated the first disease-specific HR-QOL assessment tool for adults with GTS (Gilles de la Tourette Syndrome-Quality of Life Scale, GTS-QOL). This paper presents the translation, adaptation and validation of the GTS-QOL for young Italian patients with GTS.

**Methods:** A three-stage process involving 75 patients with GTS recruited through three Departments of Child and Adolescent Neuropsychiatry in Italy led to the development of a 27-item instrument (Gilles de la Tourette Syndrome-Quality of Life Scale in children and adolescents, C&A-GTS-QOL) for the assessment of HR-QOL through a clinician-rated interview for 6–12 year-olds and a self-report questionnaire for 13–18 year-olds.

**Results:** The C&A-GTS-QOL demonstrated satisfactory scaling assumptions and acceptability. Internal consistency reliability was high (Cronbach’s alpha > 0.7) and validity was supported by interscale correlations (range 0.4–0.7), principal-component factor analysis and correlations with other rating scales and clinical variables.

**Conclusions:** The present version of the C&A-GTS-QOL is the first disease-specific HR-QOL tool for Italian young patients with GTS, satisfying criteria for acceptability, reliability and validity.

## 1. Introduction

Gilles de la Tourette syndrome (GTS) is a chronic neuropsychiatric disorder characterised by multiple motor tics and at least one vocal/phonic tic for the duration of one year, with onset in childhood [1]. Tics

ISSN 0953-4180/13/$27.50 © 2013 – IOS Press and the authors. All rights reserved are defined as sudden, rapid, recurrent, non-rhythmic, stereotyped movements or vocalizations. Prevalence rates show wide variability, but recent studies suggest a prevalence in youths aged 5–18 of about 1%, with a male:female ratio of 3:1 [2].

Although motor and phonic tics are the hallmark of GTS, this condition has been progressively recognised as a complex disorder associated with a wide spectrum of behavioural problems. Around 90% of patients seen in specialist clinics present with co-morbid behavioural difficulties, ranging from complex tic-like symptoms (self-injurious behaviours, non obscene socially inappropriate behaviours, copro-, echo- and paliphenomena) to attention deficit hyperactivity disorder (ADHD), obsessive-compulsive disorder (OCD), mood disorders, impulse control disorders and personality disorders [3,4].

Tics are associated with significant impairment and personal distress. Moreover, co-morbid behavioural problems can lead to social difficulties and impulse dyscontrol in young patients [5]. Both tics and GTS-associated disorders (i.e. ADHD, OCD) are potentially socially disabling and can bear a detrimental impact on health-related quality of life (HR-QOL) [6–11]. A recent study by Eddy et al. [10] showed that patients without co-morbidities ("Pure-GTS") presented with lower impact in their quality of life than GTS subjects with OCD and/or ADHD.

HR-QOL is emerging as a critical measure of clinical outcome, as it takes into account the patient's own subjective view on the impact of the medical condition on his overall well-being. Disease-specific HR-QOL measures have been developed for several neurological conditions, and the first disease-specific HR-QOL assessment tool for adult patients with GTS (Gilles de la Tourette Syndrome-Quality of Life Scale, GTS-QOL) has recently been developed and validated [12].

We report the development and full validation of the first disease-specific HR-QOL assessment tool translated and validated for Italian young patients with GTS: the Gilles de la Tourette Syndrome-Quality of Life Scale for children and adolescents (C&A-GTS-QOL).

## 2. Methods

The Italian version of C&A-GTS-QOL was developed in three stages. The study was approved by the local Ethics Committee and written informed consent was obtained from each subject prior to enrolment into the development and validation protocols.

Initially, forward-backward procedure was applied to translate the GTS-QOL [12] from English into Italian (Stage 1: scale translation). A professional translator translated the questionnaire into Italian ("forward translation") and this version was backward translated into English ("backward translation") by two blind professional translators The authors compared the two translated versions with the original English version, to yield the linguistic validation of the provisional questionnaire in Italian. In order to check the Italian population's understanding and interpretation of the translated items, the questionnaire was pre-tested on 10 patients with GTS (clinical sample). The results were discussed between experts and patients. This process led to a new Italian version of the GTS-QOL.

Secondly, this scale was adapted for children and adolescents and the C&A-GTS-QOL was created through the following steps (Stage 2: scale adaptation):

- Four clinicians with expertise in the management of children and adolescents with GTS independently suggested how to simplify and rephrase items that they considered to be confusing for youths, and how to change the context of items referring to adult life (e.g. job) to fit with a child's routine (e.g. school).
- The experts' opinions for each of the 27 items were compared and discussed with young patients with GTS and the questionnaire was modified accordingly.
- The questionnaire was administered to 20 children (through interview) and 20 adolescents (through selfreport questionnaire) randomly selected from a nonclinical sample (school population). The recruited subjects were asked to comment on the comprehensibility of the items and to put them into their own words.
- Wording adjustments were made by the same expert clinicians.
- To identify any further confusing items or words, the adjusted questionnaire was administered to 75 youths from a school population in both the interview version (48 children) and self-report version (27 adolescents). Items rated as confusing by over 2% of the total sample were reworded or replaced.

Thirdly, the psychometric properties of the C&A-GTS-QOL were examined in an independent sample of 75 young patients with GTS (60 males; age range:

6.8–18.3 years) recruited during 2009–2010 (Stage 3: scale evaluation). This sample was recruited from three Italian specialist centres: the Child and Adolescent Neuropsychiatry Unit at University of Insubria, Varese ($n = 21$), the Department of Child Neuropsychiatry at University of Catania ($n = 30$), and the Department of Child Neuropsychiatry at University of Rome ($n = 24$). Participants were aged 6–18 years, had no learning disabilities (IQ < 70) or other neurological conditions, and met DSM-IV-TR criteria for the diagnosis of GTS. All participants were evaluated by neuropsychiatrists with substantial expertise in management of GTS, who performed neurological examination, clinical interview and cognitive evaluation. The clinical interview included the National Hospital Interview Schedule for GTS (NHIS-TS), a detailed semi-structured interview schedule which covers personal and family histories and demographic details [13], and the Diagnostic Confidence Index (DCI), which rates the lifetime likelihood of having GTS [14]. The KIDDIE-SADS-PL, a semi-structured diagnostic interview designed to assess current and past episodes of psychopathology in children and adolescents according to DSM-III-R and DSM-IV criteria [15] was used to validate the NHIS-TS diagnosis of various GTS-associated disorders, such as ADHD and OCD. The severity of tic symptoms was assessed using the Yale Global Tic Severity Scale (YGTSS) [16] whilst the severity of obsessive-compulsive symptoms was rated using the Children's Yale-Brown Obsessive Compulsive Scale (CY-BOCS) [17]. All participants were asked to complete a booklet which included the C&A-GTS-QOL, the Child Depression Inventory (CDI) [18] and the Multidimensional Anxiety Scale for Children (MASC) [19]. The CDI is a 27-item self-report instrument that assesses depression symptoms in children and adolescents aged 7–17 years, while the MASC is a 39-item self-report scale that robustly represents the factor structure of anxiety in children aged 8–18 years.

Standard statistical methods were used to assess the psychometric properties of the C&A-GTS QOL. We examined scaling assumptions (equivalence of item response option frequency distributions, item mean scores, item standard deviations, item-total correlations, principal-component factor analysis); acceptability (distributions of subscale total scores, floor and ceiling effects); reliability (internal consistency reliability: Cronbach's alpha); and validity (interscale correlations, convergent and discriminant validity, and group differences validity). The convergent validity of the final questionnaire was examined by measuring the association between C&A-GTS-QOL subscores and demographic and clinical variables, including age, disease duration, ratings of tic severity (YGTSS scores), OCD symptoms (CY-BOCS scores), depression (CDI), anxiety (MASC). Associations between scores were tested with Spearman rank correlations because of the ordinal nature of the scales. All statistical analyses were performed using SPSS for Windows.

Table 1
Demographic and clinical characteristics of the GTS sample participating in stage 3 (scale evaluation)

| Characteristics | Overall population ($n = 75$) |
|---|---|
| Male gender, n (%) | 60 (80.0) |
| Age (yr), mean (sd) | 12.4 (3.2) |
| Age at onset of GTS (yr), mean (sd) | 6.7 (2.4) |
| Duration of GTS (yr), mean (sd) | 2.0 (2.0) |
| Age at diagnosis of GTS (yr), mean (sd) | 10.3 (2.7) |
| 'Pure' GTS, n (%) | 33 (44.0) |
| Presence of OCD, n (%) | 25 (33.3) |
| Presence of ADHD, n (%) | 6 (8.0) |
| Presence of ADHD + OCD, n (%) | 11 (14.7) |
| Presence of coprolalia, n (%) | 18 (24.0) |
| Presence of copropraxia, n (%) | 9 (12.0) |
| Presence of echolalia, n (%) | 20 (26.7) |
| Presence of echopraxia, n (%) | 5 (6.7) |
| Presence of palilalia, n (%) | 11 (14.7) |
| Patients with pharmacotherapy, n (%) | 40 (53.3) |
| DCI score, mean (sd) | 85.6 (23.8) |
| YGTSS total score, mean (sd) | 44.3 (20.5) |
| CY-BOCS total score, mean (sd) | 11.4 (10.0) |
| CDI score, mean (sd) | 9.5 (6.9) |
| MASC total score, mean (sd) | 49.5 (10.2) |

Abbreviations. GTS = Gilles de la Tourette Syndrome; OCD = obsessive-compulsive disorder; ADHD = attention deficit hyperactivity disorder; DCI = Diagnostic Confidence Index; YGTSS = Yale Global Tic Severity Scale; CY-BOCS = Children's Yale-Brown Obsessive Compulsive Scale; CDI = Child Depression Inventory; MASC = Multidimensional Anxiety Scale for Children.

## 3. Results

The final C&A-GTS-QOL consisted of 27 items and four subscales (psychological, physical, obsessive-compulsive and cognitive) in two age-adjusted versions (see Appendix). The first version is an interview to be administered by a qualified clinician, for youths aged 6–12 years. The second is a self-administered questionnaire for adolescents aged 13–18 years. Each item is rated across five response options: 'Never', 'Rarely', 'Sometimes', 'Often', 'Always'.

The C&A-GTS-QOL was evaluated with respect to scaling assumptions, acceptability, reliability and validity, in a sample of 75 young patients with GTS. The demographic and clinical characteristics of the GTS sample are shown in Table 1.

Table 2
Score distributions and reliability of the C&A-GTS-QOL

| | Psychological subscale | Physical subscale | Obsessive-compulsive subscale | Cognitive subscale |
|---|---|---|---|---|
| *Scaling assumptions* | | | | |
| Range of item mean scores | 0.7–1.4 | 0.4–1.4 | 0.5–1.3 | 0.7–1.4 |
| Range of item SD | 1.0–1.3 | 0.9–1.3 | 1.0–1.3 | 1.0–1.2 |
| Range of corrected item-total correlations | 0.5–0.7 | 0.3–0.5 | 0.3–0.7 | 0.3–0.6 |
| *Acceptability* | | | | |
| Mean (SD) | 11.7 (9.0) | 6.1 (4.6) | 4.8 (3.9) | 4.1 (3.1) |
| Median | 10.0 | 5. | 4. | 4. |
| Range of scores | 0–42 | 0–22 | 0–17 | 0–1 |
| Skewness/SE skewness | 0.7/0.3 | 0.9/0.3 | 0.6/0.3 | 0.7/0.3 |
| Floor/ceiling effects, % | 0.3/0.5 | 0.8/0.5 | 0.0/0.5 | 0.0/0.5 |
| *Reliability* | | | | |
| Internal consistency (Cronbach's alpha) | 0.9 | 0.7 | 0.7 | 0.7 |

Abbreviations. C&A-GTS-QOL = Gilles de la Tourette Syndrome-Quality of Life Scale for Children and Adolescents.

Table 3
Principal-component factor analysis of the 27-item C&A-GTS-QOL (oblimin rotated solution; loading $\geqslant$ 0.50 are shown)

| | Factor 1: psychological | Factor 2: physical | Factor 3: obsessive-compulsive | Factor 4: cognitive |
|---|---|---|---|---|
| Depressed mood | 0.87 | | | |
| Lack of control over own life | 0.45 | | | |
| Loneliness/isolation | 0.67 | | | |
| Lack of self-confidence | 0.65 | | | |
| Frustration | 0.59 | | | |
| Lack of social support | 0.67 | | | |
| Anxiety | 0.84 | | | |
| Difficulty seeing friends | 0.56 | | | |
| Mood switches | 0.70 | | | |
| Temper dyscontrol | 0.45 | | | |
| Restlessness | 0.73 | | | |
| Embarrassing gestures | | 0.83 | | |
| Difficulty in daily life activities | | 0.24 | | |
| Involuntary swearing | | 0.84 | | |
| Pain or incurie | | 0.60 | | |
| Movement dyscontrol | | 0.40 | | |
| Phonic tics | | 0.28 | | |
| Difficulty taking part in social activities | | −0.04 | | |
| Repeating words | | | 0.67 | |
| Copying people | | | 0.62 | |
| Concerns about poor health | | | 0.61 | |
| Unpleasant thoughts | | | 0.73 | |
| Repeating actions | | | 0.72 | |
| Memory problems | | | | 0.67 |
| Difficulty concentrating | | | | 0.76 |
| Losing important things | | | | 0.70 |
| Difficulty finishing tasks | | | | 0.68 |
| % of variance explained | 33.99 | 7.96 | 6.79 | 6.34 |

Abbreviations. C&A-GTS-QOL = Gilles de la Tourette Syndrome-Quality of Life Scale for Children and Adolescents.

### 3.1. Acceptability

For each scale, all items/response options were endorsed, with only one exception (item 26: 'Difficulty taking part in social activities'). The frequency distributions of the items were relatively symmetrical and not unduly skewed (low floor and ceiling effects), and item mean scores and standard deviations were similar (Table 2). Principal-component factor analysis (oblimin rotated solution) of the 27 items showed no cross-loadings, thus supporting the grouping of items into four subscales and their summing in each subscale to produce a total score (Table 3). The resulting four-factor model accounted for 55.08% of the overall variance. The pattern of score distributions is presented in Table 2.

Table 4
Spearman correlation coefficients between C&A-GTS-QOL scores and measures of health status and psychological well-being

| | Total | Psychological subscale | Physical subscale | Obsessive-compulsive subscale | Cognitive subscale |
|---|---|---|---|---|---|
| Age | 0.01 | 0.10 | 0.22 | 0.11 | 0.07 |
| Disease duration | 0.08 | 0.11 | 0.06 | 0.15 | 0.03 |
| YGTSS | | | | | |
| Motor tics | 0.11 | 0.10 | 0.22 | 0.15 | 0.11 |
| Vocal tics | 0.25** | 0.27** | 0.23** | 0.28** | 0.02 |
| Impairment | 0.34** | 0.33** | 0.29** | 0.31** | 0.16 |
| CY-BOCS | | | | | |
| Obsessions | 0.13 | 0.10 | 0.17 | 0.23** | 0.13 |
| Compulsions | 0.12 | 0.06 | 0.19 | 0.29** | 0.10 |
| Depression (CDI) | 0.65* | 0.63* | 0.51* | 0.49* | 0.51* |
| Anxiety (MASC) | 0.56* | 0.53* | 0.56* | 0.43* | 0.29** |

$^{*}p < 0.001$; $^{**}p < 0.05$.
Abbreviations. C&A-GTS-QOL = Gilles de la Tourette Syndrome-Quality of Life Scale for Children and Adolescents; YGTSS = Yale Global Tic Severity Scale; CY-BOCS = Children's Yale-Brown Obsessive Compulsive Scale; CDI = Child Depression Inventory; MASC = Multidimensional Anxiety Scale for Children.

### 3.2. *Reliability*

For all subscales, corrected item-total correlations considerably exceeded the recommended criterion of 0.4 [20] and all internal consistency reliability estimates (Cronbach's alpha) exceeded 0.7 (Table 2).

### 3.3. *Validity*

The content validity of the final subscales was assessed by an expert panel. Internal construct validity was supported by the moderate correlation between the subscales (range 0.4–0.7), implying that the GTS-QOL subscales measure related but relatively different health constructs. Convergent and discriminant construct validity was supported by correlations with other scales and variables that were shown to influence HR-QOL in GTS (e.g. depression and anxiety) [10]. Table 4 shows the Spearman correlation coefficients between the GTS-QOL subscales and measures of health status and psychological well-being. Finally, there were no differences between scores for men and women.

## 4. Discussion

HR-QOL has consistently been shown to be impaired in young patients with GTS. However, until recently there has been no specific HR-QOL scale for children and adolescent with GTS. Previous studies on HR-QOL in this patient population have all used generic HR-QOL instruments. In their study involving 59 patients with GTS (age range 8–17 years), Storch et al. [7] administered a generic HR-QOL instrument, the Pediatric Quality of Life inventory and Parent Proxy form (PedsQL) and found that the main predictors of low HR-QOL scores were tic severity and 'internalizing'/'externalizing' behavioural symptoms. Bernard et al. [8] found that co-morbid ADHD (inattentive type) and OCD were predictors of poor HR-QOL in 56 patients with GTS aged 5–17 years, using the TNO-AZL Children's Quality of Life scale-Parent Form (TACQOL-PF). Similar results were found in another study of 57 youths with GTS (age range 8-17 years) using the PedsQL [9]. More recently, Eddy et al. [10] administered the Youth Quality of Life Instrument-Research Version (YQL-RV) in a clinical sample of 50 youths with GTS (age range 11–17 years) and found that different areas of HR-QOL were affected in relation to different clinical presentations of the 'GTS behavioural spectrum' [3]. Although the use of generic instruments has the advantage of allowing comparison between different disease groups [21], it has significant limitations in neuropsychiatric disorders characterised by specific motor and behavioural symptoms. With regards to GTS, generic instruments do not address and are unlikely to be sensitive to tics and tic-related symptoms or their specific impacts on HR-QOL. Consequently, the generic PedsQL, TACQOL-PF and YQL-RV are likely to underestimate or misrepresent the subjective perception of health problems in GTS. This prompted the development of a disease-specific HR-QOL scale for young patients with GTS.

This study presents the development and validation of a disease-specific scale for the subjective assessment of HR-QOL in young Italian patients with GTS, distinguishing the perceived problems of children aged 6–12 years from those of adolescents aged 13–18 years. Following translation and linguistic adaptation of the 27-item GTS-QOL [12], we developed two different versions of the C&A-GTS-QOL: an interview to be administered by qualified clinicians to 6–12 year old children and a self-administered questionnaire for adolescents (13–18 years). When testing the psychometric properties of the C&A-GTS-QOL in a large sample of young patients with GTS, we found that our overall results were in line with previous studies. According to C&A-GTS-QOL standardised mean scores, HR-QOL perception was mainly affected by physical disability [7] and OCD symptoms [10], followed by psychological problems [6,11] and cognitive difficulties. Notably, the four subscales of the C&A-GTS-QOL cover all aspects that have been shown to bear an impact on HR-QOL in GTS.

The results of our validation study suggest that the translation and adaptation of the GTS-QOL into the A&C-GTS-QOL is well suited for use in the Italian population of young patients with GTS. Scaling assumptions were satisfactory and acceptability was good. Internal consistency reliability, as measured by Cronbach's alpha coefficients, ranged from 0.7 for the physical, obsessive-compulsive and cognitive subscales to 0.9 for psychological subscale, suggesting that these subscales actually measure the same construct and support the construct validity. Convergent and discriminant validity showed a sensible pattern of correlations between other variables and clinical scales addressing HR-QOL.

Our study has limitations. The comprehensibility of scale items was judged by a small non-clinical sample (school population), randomly recruited in a limited area. While we were mindful to include patients with wide phenotype variability in the evaluation of the scale, it is likely that patients with more severe phenotype were over-represented in our sample, which may not be representative of the community GTS population. Finally, it is important to stress that the development of the scale was based on and targeted at the Italian population. Thus, the psychometric properties of the C&A-GTS-QOL scale need further testing in different clinic and community populations.

The C&A-GTS-QOL is the first disease-specific HR-QOL scale for Italian children and adolescents with GTS; it will provide a useful additional subjective measure complementing available objective clinical rating scales. Moreover, the C&A-GTS-QOL is user-friendly, takes only about 15 minutes to complete and could have useful applications as a patient-report outcome measure in clinical trials involving young patients with GTS. Ongoing research includes the back-translation of the Italian version of C&A-GTS-QOL into English language to enable the use of this new instrument in other Countries. Future research will also allow insightful cross-cultural comparisons of perceived HR-QOL in young populations with GTS.

## Acknowledgments

The authors are grateful to Tourettes Action-UK and ESSTS for continuing support. This work was partially funded by COST Action BM0905.

## Appendix

### Sindrome di Gilles de la Tourette – Scala sulla qualità della vita 6–12 (GTS-QOL-C&A 6–12)

Avere problemi di salute può interferire con la qualità della vita di una persona in modi diversi. Questo questionario valuta come la malattia interferisce con il tuo benessere. Per favore, traccia una croce nella casella corrispondente alla risposta che meglio descrive l'entità di ciascun problema. Questa lista include problemi che potresti non aver mai avuto.

| Nelle ultime 4 settimane. . . | MAI | RARAMENTE | QUALCHE VOLTA | SPESSO | SEMPRE |
|---|---|---|---|---|---|
| 1. Hai avuto difficoltà a controllare i tuoi movimenti? | □ | □ | □ | □ | □ |
| 2. Hai avuto difficoltà a compiere le attività scolastiche o sportive che preferisci? | □ | □ | □ | □ | □ |
| 3. Hai avuto dolore o ti sei fatto male per i tuoi tic? | □ | □ | □ | □ | □ |
| 4. Sei stato disturbato da rumori che non riuscivi a smettere di fare? | □ | □ | □ | □ | □ |
| 5. Ti sei preoccupato per aver detto brutte parole senza volere? | □ | □ | □ | □ | □ |
| 6. Ti sei preoccupato per aver fatto gesti volgari senza volerlo? | □ | □ | □ | □ | □ |
| 7. Ti è capitato di ripetere alcune parole più e più volte? | □ | □ | □ | □ | □ |
| 8. Hai dovuto ripetere parole o gesti di altre persone senza volere? | □ | □ | □ | □ | □ |
| 9. Hai dovuto ripetere alcune cose più e più volte nello stesso modo (es. controllare, toccare)? | □ | □ | □ | □ | □ |
| 10. Ti è capitato di avere brutti pensieri o brutte immagini nella tua mente ? | □ | □ | □ | □ | □ |
| 11. Hai avuto difficoltà di concentrazione? | □ | □ | □ | □ | □ |
| 12. Hai avuto problemi di memoria? | □ | □ | □ | □ | □ |
| 13. Hai perso o messo fuori posto cose importanti (es. libri, quaderni, chiavi, cellulare)? | □ | □ | □ | □ | □ |
| 14. Hai avuto difficoltà a finire un compito una volta iniziato? | □ | □ | □ | □ | □ |
| 15. Ti sei sentito male? | □ | □ | □ | □ | □ |
| 16. Sei stato triste o depresso? | □ | □ | □ | □ | □ |
| 17. Ti è capitato di diventare improvvisamente triste o improvvisamente allegro senza motivo? | □ | □ | □ | □ | □ |
| 18. Hai smesso di fare qualcosa perché pensavi di non riuscirci? | □ | □ | □ | □ | □ |
| 19. Ti sei sentito scontento? | □ | □ | □ | □ | □ |
| 20. Ti sei sentito agitato? | □ | □ | □ | □ | □ |
| 21. Hai avuto difficoltà a controllare la tua rabbia? | □ | □ | □ | □ | □ |
| 22. Ti sei sentito come se non fossi in grado di controllare ciò che fai? | □ | □ | □ | □ | □ |
| 23. Ti sei arrabbiato quando non sei riuscito a fare qualcosa? | □ | □ | □ | □ | □ |
| 24. Hai sentito il bisogno di maggiore aiuto o incoraggiamento da parte di altre persone? | □ | □ | □ | □ | □ |
| 25. Hai avuto difficoltà a stare insieme ai tuoi amici? | □ | □ | □ | □ | □ |
| 26. Hai avuto difficoltà nelle uscite fuori casa con gli altri (es. cinema, feste)? | □ | □ | □ | □ | □ |
| 27. Ti sei sentito solo o isolato? | □ | □ | □ | □ | □ |

Per favore, indica quanto ti senti contento della tua vita in questo momento mettendo una croce sulla linea da 0 a 100:

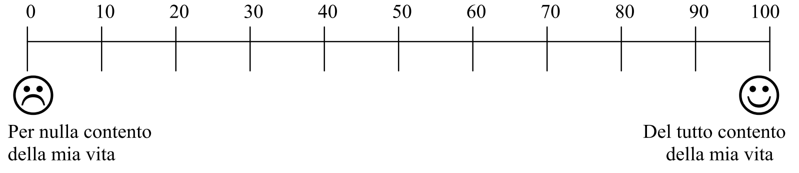

Grazie per aver compilato questo questionario!

## Sindrome di Gilles de la Tourette – Scala sulla qualità della vita 13–18 (GTS-QOL-C&A 13–18)

Avere problemi di salute può interferire con la qualità della vita di una persona in modi diversi. Questo questionario valuta come la malattia interferisce con il tuo benessere. Per favore, traccia una croce nella casella corrispondente alla risposta che meglio descrive l'entità di ciascun problema. Questa lista include problemi che potresti non aver mai avuto.

| Nelle ultime 4 settimane . . . | MAI | RARAMENTE | QUANCHE VOLTA | SPESSO | SEMPRE |
|---|---|---|---|---|---|
| 1. Hai avuto difficoltà a controllare i tuoi movimenti? | □ | □ | □ | □ | □ |
| 2. Hai avuto difficoltà nello svolgimento delle attività quotidiane o degli svaghi che preferisci (cucinare, scrivere)? | □ | □ | □ | □ | □ |
| 3. Hai avuto dolore o danni fisici conseguenti ai tuoi tic? | □ | □ | □ | □ | □ |
| 4. Hai avuto problemi dovuti a rumori che non riuscivi a smettere di fare? | □ | □ | □ | □ | □ |
| 5. Ti sei preoccupato per aver usato parolacce che in realtà non volevi dire? | □ | □ | □ | □ | □ |
| 6. Ti sei preoccupato per aver fatto gesti imbarazzanti (es. gesti volgari)? | □ | □ | □ | □ | □ |
| 7. Hai dovuto ripetere alcune parole molte volte? | □ | □ | □ | □ | □ |
| 8. Hai dovuto ripetere cose che altre persone hanno detto o fatto (es. imitare persone)? | □ | □ | □ | □ | □ |
| 9. Hai dovuto ripetere alcune azioni più e più volte nello stesso modo (es. controllare, toccare)? | □ | □ | □ | □ | □ |
| 10. Hai avuto pensieri spiacevoli o immagini che si insinuavano nella tua mente? | □ | □ | □ | □ | □ |
| 11. Hai avuto difficoltà di concentrazione? | □ | □ | □ | □ | □ |
| 12. Hai avuto problemi di memoria? | □ | □ | □ | □ | □ |
| 13. Hai perso o messo fuori posto cose importanti (es. chiavi, cellulare)? | □ | □ | □ | □ | □ |
| 14. Hai rinunciato a finire un compito una volta iniziato? | □ | □ | □ | □ | □ |
| 15. Non ti sei sentito in forma? | □ | □ | □ | □ | □ |
| 16. Sei stato triste o depresso? | □ | □ | □ | □ | □ |
| 17. Ti è capitato di passare rapidamente dall'essere allegro all'essere triste? | □ | □ | □ | □ | □ |
| 18. Hai avuto mancanza di fiducia in te stesso? | □ | □ | □ | □ | □ |
| 19. Ti sei sentito ansioso? | □ | □ | □ | □ | □ |
| 20. Ti sei sentito agitato? | □ | □ | □ | □ | □ |
| 21. Hai avuto difficoltà a controllare la tua collera? | □ | □ | □ | □ | □ |
| 22. Ti sei sentito come se non avessi il controllo della tua vita? | □ | □ | □ | □ | □ |
| 23. Ti sei sentito frustrato? | □ | □ | □ | □ | □ |
| 24. Hai sentito la necessità di un maggiore aiuto o supporto da parte di altre persone? | □ | □ | □ | □ | □ |
| 25. Hai avuto difficoltà a frequentare i tuoi amici? | □ | □ | □ | □ | □ |
| 26. Hai avuto difficoltà a prendere parte ad attività sociali (es. mangiare fuori)? | □ | □ | □ | □ | □ |
| 27. Ti sei sentito solo o isolato? | □ | □ | □ | □ | □ |

Per favore, indica quanto ti senti soddisfatto della tua vita in questo momento mettendo una croce sulla linea da 0 a 100:

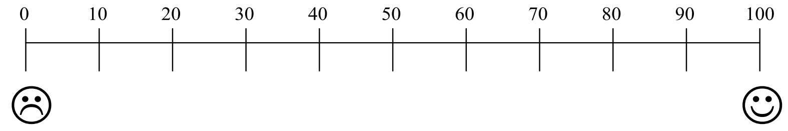

Estremamente insoddisfatto della mia vita

Estremamente soddisfatto della mia vita

Grazie per aver compilato questo questionario!
